# CpG Methylation Controls Reactivation of HIV from Latency

**DOI:** 10.1371/journal.ppat.1000554

**Published:** 2009-08-21

**Authors:** Jana Blazkova, Katerina Trejbalova, Françoise Gondois-Rey, Philippe Halfon, Patrick Philibert, Allan Guiguen, Eric Verdin, Daniel Olive, Carine Van Lint, Jiri Hejnar, Ivan Hirsch

**Affiliations:** 1 Institut National de la Santé et de la Recherche Médicale (INSERM), UMR891, Centre de Recherche en Cancérologie de Marseille; and Institut Paoli-Calmettes, Marseille, France; 2 Université Méditerranée, Marseille, France; 3 Institute of Molecular Genetics, Academy of Sciences of the Czech Republic, Prague, Czech Republic; 4 Laboratory of Molecular Virology, Institute for Molecular Biology and Medicine (IBMM), University of Brussels (ULB), Gosselies, Belgium; 5 Department of Virology, Alphabio Laboratory, Marseilles, France; 6 Department of Infectious Diseases, Hôpital Ambroise Paré, Marseilles, France; 7 Gladstone Institute of Virology and Immunology, San Francisco, California, United States of America; Northwestern University, United States of America

## Abstract

DNA methylation of retroviral promoters and enhancers localized in the provirus 5′ long terminal repeat (LTR) is considered to be a mechanism of transcriptional suppression that allows retroviruses to evade host immune responses and antiretroviral drugs. However, the role of DNA methylation in the control of HIV-1 latency has never been unambiguously demonstrated, in contrast to the apparent importance of transcriptional interference and chromatin structure, and has never been studied in HIV-1-infected patients. Here, we show in an *in vitro* model of reactivable latency and in a latent reservoir of HIV-1-infected patients that CpG methylation of the HIV-1 5′ LTR is an additional epigenetic restriction mechanism, which controls resistance of latent HIV-1 to reactivation signals and thus determines the stability of the HIV-1 latency. CpG methylation acts as a late event during establishment of HIV-1 latency and is not required for the initial provirus silencing. Indeed, the latent reservoir of some aviremic patients contained high proportions of the non-methylated 5′ LTR. The latency controlled solely by transcriptional interference and by chromatin-dependent mechanisms in the absence of significant promoter DNA methylation tends to be leaky and easily reactivable. In the latent reservoir of HIV-1-infected individuals without detectable plasma viremia, we found HIV-1 promoters and enhancers to be hypermethylated and resistant to reactivation, as opposed to the hypomethylated 5′ LTR in viremic patients. However, even dense methylation of the HIV-1 5′LTR did not confer complete resistance to reactivation of latent HIV-1 with some histone deacetylase inhibitors, protein kinase C agonists, TNF-α, and their combinations with 5-aza-2deoxycytidine: the densely methylated HIV-1 promoter was most efficiently reactivated in virtual absence of T cell activation by suberoylanilide hydroxamic acid. Tight but incomplete control of HIV-1 latency by CpG methylation might have important implications for strategies aimed at eradicating HIV-1 infection.

## Author Summary

Despite the potency of highly active antiretroviral therapy (HAART) to decrease the HIV-1 load and to reduce mortality due to HIV-1 infection, HIV-1 establishes latent infection resistant to host immune responses and antiretroviral therapy. HIV-1 latency is thus the main obstacle to the eradication of the virus from infected patients. CpG methylation is a mechanism which contributes to transcriptional silencing. The role of proviral DNA methylation in HIV-1 latency has not been clearly demonstrated and has never been studied in HIV-1-infected patients. We found in an *in vitro* model and in HIV-1-infected patients that CpG methylation of the HIV-1 promoter is important for the maintenance but not for the establishment of HIV-1 latency. We show that tight control of HIV-1 latency by CpG methylation could be a key barrier to purging the reservoir of latently infected cells in infected individuals. Although our study shows the difficulty in reactivation of HIV-1 with the heavily methylated promoter/enhancer sequences from latently infected cells, it also indicates that addition of some histone deacetylase inhibitors (namely suberoylanilide hydroxamic acid, SAHA) and cytosine methylation inhibitors would represent an important part of HAART protocols in the future.

## Introduction

The current protocols of highly active antiretroviral therapy (HAART) are efficient in decreasing the HIV-1 load below the limit of detection, reducing mortality due to HIV-1 infection. Despite the potency of HAART, however, HIV-1 establishes latent infection in a reservoir of resting memory CD4^+^ T cells, which escapes host immune responses and antiretroviral therapy. HIV-1 latency is thus the main obstacle to the eradication of the virus from infected patients [1]–[5].

Transcriptional shutdown and multistep formation of restrictive chromatin at long terminal repeats (LTR) are two interconnected events leading to the latent state of HIV-1 provirus. HIV-1 LTR-driven transcription is silenced in the absence of cellular transcription initiation factors NF-κB and NFAT [6],[7] or in the presence of repressors such as CBF-1 and YY1 [8],[9]. Low levels of the Tat transactivator [10] or the Tat-activated elongation factor P-TEFb [11], and sustained production of prematurely terminated RNA transcripts from the HIV-1 promoter [12],[13] are hallmarks of HIV-1 latency. At the level of chromatin, entry of HIV-1 into latency requires recruitment of the histone deacetylase type 1 (HDAC-1) [8],[9],[14], histone methyltransferase Suv39H1, and heterochromatin protein HP1 [15],[16] to the chromatin around the HIV-1 LTR. It was suggested that in contrast to the handful of factors triggering HIV-1 latency, NF-κB alone has the potential to reactivate HIV-1 from its latent state, and it might be a master factor in this process [7]. However, more recent reports show that HIV-1 can be activated in an NF-κB-independent way by transcription factor ΔVII-Ets-1, without causing significant T cell activation [17], and that Lck and NFAT, but not NF-κB, are required for optimal latent virus reactivation in primary memory T cells [18]. Transcriptional reactivation is accompanied by changes in the local chromatin structure and by the recruitment of chromatin remodeling factors such as SWI/SNF [19],[20] and histone acetyl transferases such as CBP and p300 [21],[22].

CpG methylation of retroviral promoter sequences is another transcriptional silencing mechanism which contributes to a difficulty of access of transcription factors to the target DNA. Not surprisingly, CpG methylation of retroviral promoter and enhancer sequences situated in the 5′ LTR has been correlated with silencing of various retroviruses, both infectious and endogenous, as shown for human T-cell leukemia virus type-1 [23],[24], Moloney murine leukemia virus [25],[26], Rous sarcoma virus [27],[28], and human endogenous retroviruses of the H, K, and W families [29],[30]. The role of proviral DNA methylation in HIV-1 latency is not clear. CpG methylation of the HIV-1 promoter inhibits transcription of *in vitro*-methylated plasmids transfected into cells [31],[32], and it has been suggested as a mechanism for the maintenance of HIV-1 latency in long-term-infected U937 cells [33] and in latently infected ACH-2 cell line [34], both cell systems containing silencing mutations in the HIV-1 promoter. In contrast to the previously described systems, we and others have shown that transcriptional suppression of wild-type HIV-1 promoter is not accompanied by CpG methylation of the 5′ LTR [35]–[37].

Here, we analyzed the relation of CpG methylation of the HIV-1 5′ LTR to transcriptional suppression and reactivation of HIV-1 provirus in a model consisting of Jurkat clonal cell lines harboring a latent HIV-1 LTR–driven retroviral vector [38],[39]. We compared our findings obtained in this model with the relationships between the methylation pattern, latency, and reactivation of latent HIV-1 provirus in CD4^+^ T cells of long-term aviremic and viremic patients.

## Results

### Hypomethylation of the HIV-1 5′ LTR in Latently Infected Clonal Cells of Jurkat Lines

**Figure 1:**
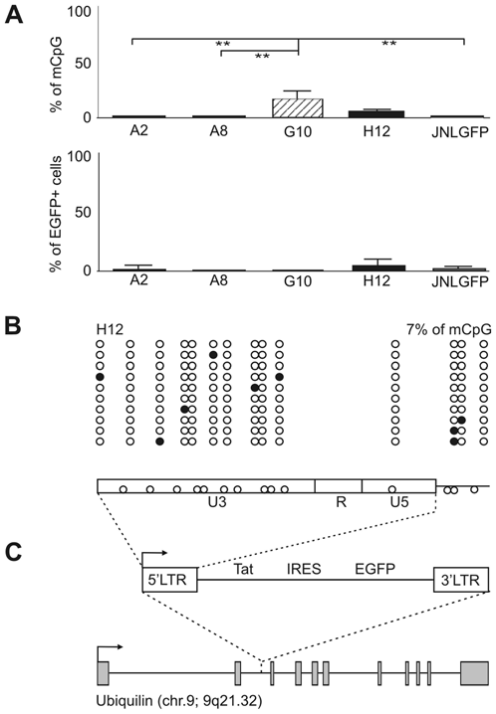
Methylation profiles of latent promoters of HIV-1 LTR-driven vectors integrated in Jurkat cells. (A) CpG methylation levels of the 5′ LTR of latent HIV-1 LTR-driven retroviral vector pEV731 coding the HIV-1 protein Tat and EGFP in Jurkat clonal cell lines A2, A8, G10, and H12, and of the latent complete EGFP-coding HIV-1 in the JNLGFP cell line. Methylation levels are presented as a mean±standard error of percentages of methylated CpGs in cloned HIV-1 promoters for each cell line. Only DNA sequences with at least 95% conversion of cytosines outside CpGs were taken into account. Levels of EGFP expression (mean±standard error) in all five cell lines are shown bellow. Significance of the difference in methylation levels between cell lines was calculated by non-parametric, two-side Mann-Whitney test. (B) Methylation pattern of the 5′ LTR in the H12 cell line. Analysis of ten promoter molecules is shown as a linear array of open circles representing nonmethylated CpG residues and closed circles representing methylated CpG residues. Shown in the rectangle representing the LTR regions U3, R, and U5 of the pEV731 vector is the distribution of CpG dinucleotides. (C) Integration site of the latent HIV-1 “mini-virus” in the H12 cell line. Gray rectangles represent the exons of *ubiquilin*. See Table S2 for the flanking sequences. mCpG, methylated CpG residues.

To study the role of CpG methylation of the HIV-1 promoter in proviral latency and during reactivation, we have chosen the Jurkat clonal cell lines A2, A8, G10, and H12 latently infected with a “mini-virus” which contained the LTR-Tat-IRES-EGFP-LTR genome [38], and the cell line JNLGFP latently infected with the complete EGFP-coding virus [40] (Figure 1). There is a single proviral copy per cell in lines A2, A8, G10, and H12 and five copies per cell in line JNLGFP (Table S1). The levels of CpG methylation in the assayed cell lines were determined by bisulfite sequencing. In the first step of this technique, sodium bisulfite converts cytosine residues in the genomic DNA to uracil, but leaves 5-methylcytosine residues unaffected. The bisulfite-treated DNA is then amplified by primers specific for the 5′ LTR, and the PCR product is cloned and sequenced. Results are interpreted based on the assumption that each cloned sequence represents an individual HIV-1 promoter, and that cytosine residues in the final sequence represent the 5′-methyl cytosines in the template DNA. Using bisulfite sequencing, we found zero or low levels of CpG methylation in the 5′ LTR (0% in A2, 0% in A8, 27±7% [mean±SEM] in G10, 7±2% in H12, and 0% in JNLGFP) (Figure 1A and B). We also found low levels of EGFP expression in all five cell lines (2±1% [mean±SEM] of EGFP-positive cells in A2, 0.4±0.1% in A8, 0.3±0.2% in G10, 5±1% in H12, and 2±1% in JNLGFP). Thus we confirmed, by means of additional clonal cell lines, that 5′ LTR methylation is not required for HIV-1 promoter silencing [35]. For the next series of experiments, we chose the intermediate H12 clonal cell line, harboring a single proviral copy integrated in the first intron of the *ubiquilin* gene (chr. 9; 9q21.32) in the sense of the *ubiquilin* transcription (Figure 1C, Table S2). Differences between distribution of the 5′-methylcytosines in the HIV-1 promoters in the H12 clonal cell line and other clonal cell lines were not statistically significant.

### Cloning of Cells Harboring Latent HIV-1 Provirus Resistant to Reactivation

**Figure 2:**
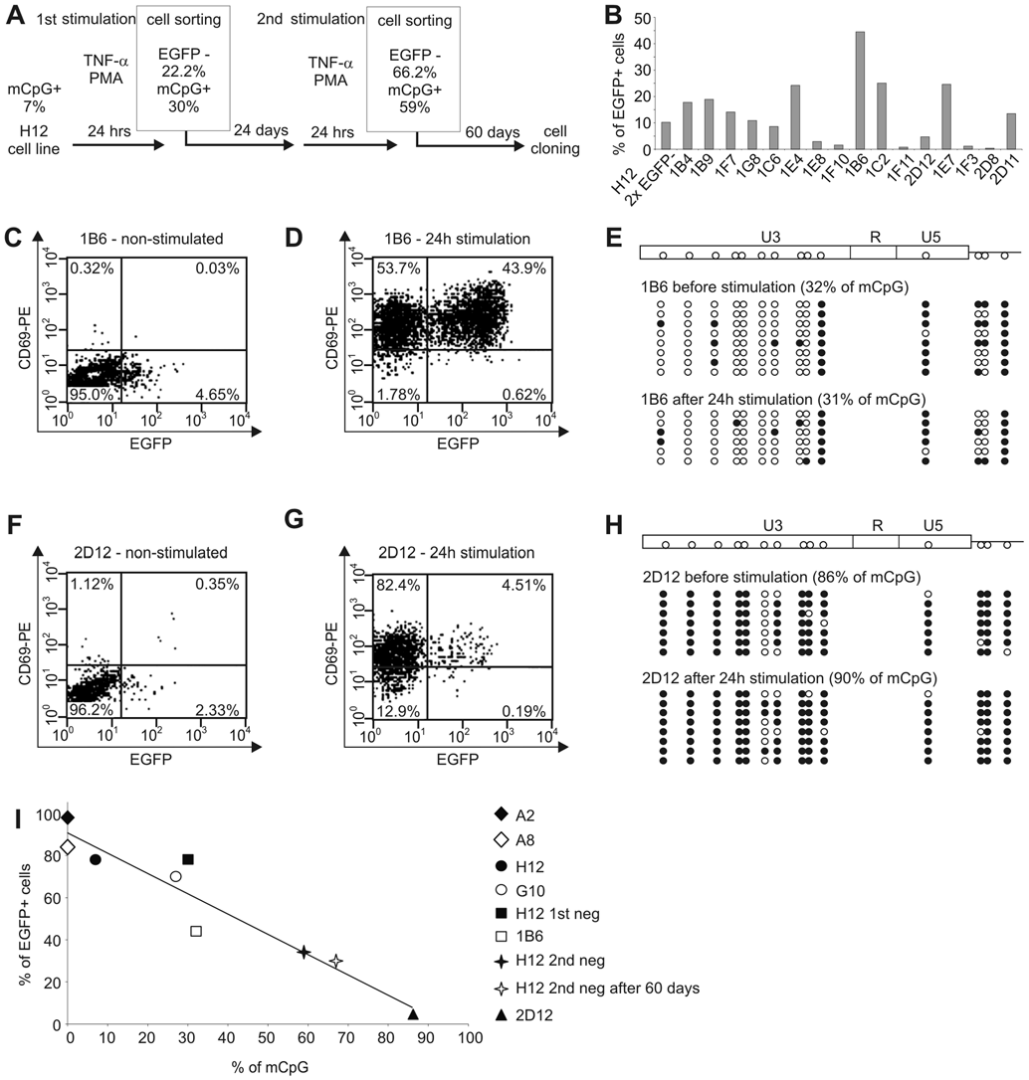
Negative correlation of CpG density in the 5′ LTR and reactivation of HIV-1 provirus. (A) Flow chart protocol showing two consecutive cycles of 24-h stimulation of H12 cells with TNF-α and PMA, followed by the first and second cell sorting of EGFP-negative cells. Negative sortings are separated by 24-day and followed by 60-day cultivation periods in the absence of any exogeneous stimulator to reach steady-state culture conditions. In the frames are shown the percentages of EGFP-negative cells determined at the moment of cell sorting and the percentages of methylated CpGs in HIV-1 promoter determined in the EGFP-negative cell population just after cell separation. (B) Reactivation levels of HIV-1 promoters in clones harboring latent HIV-1. The percentages of EGFP^+^ cells in cell clones exposed to TNF-α (10 ng/ml) and PMA (10 nM) for 24 h are shown. The cell clones were prepared by sublimit dilution of the population of H12 cells 60 days after the second sorting of EGFP-negative cells (as shown in flow chart protocol, panel A, and in Figure S1, panel I) and analyzed after the outgrowth of cell clones 20 days later. The cell culture from which the clones were derived was analyzed under the same conditions, 20 days after cell cloning (H12, 2×EGFP^−^). (C,D) Immunofluorescence of EGFP and CD69 in 1B6 clone stimulated with TNF-α (10 ng/ml) and PMA (10 nM) for 24 h (D) and in non-stimulated control (C). (E) CpG methylation profile of the 5′ LTR in non-stimulated 1B6 clone and in 1B6 clone stimulated for 24 h. (F,G) Immunofluorescence of EGFP and CD69 in 2D12 clone stimulated with TNF-α (10 ng/ml) and PMA (10 nM) for 24 h (G) and in non-stimulated control (F). (H) CpG methylation profile of the 5′ LTR in non-stimulated 2D12 clone and in 2D12 clone stimulated for 24 h. (I) Negative correlation of CpG density in the 5′ LTR of HIV-1 provirus with EGFP expression in latently infected Jurkat clonal cell lines exposed to TNF-α and PMA. Open circles, nonmethylated CpG residues; closed circles, methylated CpG residues (mCpG).

To select cells harboring latent HIV-1 provirus resistant to reactivation, we sorted the H12 cells that after two consecutive cycles of stimulation with TNF-α and PMA did not express the HIV-1 reporter gene EGFP (Figure 2A and Figure S1). This enrichment procedure resulted in increased levels of cells harboring HIV-1 provirus resistant to reactivation (from 22.2% after the first negative sorting to 66.2% after the second negative sorting), while concomitantly, CpG methylation in the 5′ LTR augmented from 7% in H12 cells before the first stimulation to 59% in H12 cells after the second negative sorting (Figure 2A and Figure S1G). To study the relation between the density of CpG methylation and the reactivation of latent HIV-1 at the level of single proviral copy, we cloned the enriched population using limiting dilution. Reactivation levels of HIV-1 provirus in the resulting 16 clones stimulated with TNF-α and PMA varied between 0.4% and 44.5% of cells (Figure 2B), providing a basis for testing the role of CpG methylation in the process.

### Tight Control of HIV-1 Latency is Related to Hypermethylation of the 5′ LTR

For further experiments, we selected the clones 1B6 and 2D12, which responded distinctly to reactivation with TNF-α and PMA (Figure 2B) and which showed low spontaneous activity of the HIV-1 promoter (Figure 2C and F). In the 1B6 clone, stimulation with TNF-α and PMA reactivated the HIV-1 provirus from latency in 44.5% of cells (Figure 2D), and 32% of CpGs in the 5′ LTR were methylated (Figure 2E). In contrast, the cell clone 2D12 displayed active HIV-1 promoter in only 4.7% of cells after stimulation with TNF-α and PMA (Figure 2G), and 86% of CpGs in the 5′ LTR were methylated (Figure 2H). Expression of early activation marker of CD4^+^ T cells CD69 on the surface of the majority of 1B6 cells (97.6%, Figure 2D) and 2D12 cells (86.9%, Figure 2G) stimulated with TNF-α and PMA shows that the weak reactivation of HIV-1 promoter was not caused by the inability of the host cells to be activated. Proportions of methylated CpGs in both clones stimulated with TNF-α and PMA remained stable for at least the next 24 h (Figure 2E and H). As shown in Figure 2I, the density of 5′ LTR CpG methylation in clonal cell lines H12, 1B6, 2D12, A2, A8, G10, and in cell cultures derived from the cell line H12 negatively correlates with the reactivation of HIV-1 promoters, as determined at the transcriptional level by quantitative RT-PCR (not shown) and at the translational level from expression of EGFP.

**Figure 3:**
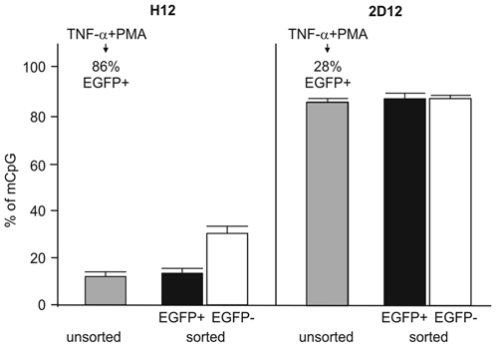
DNA methylation levels of the 5′ LTR after reactivation of HIV-1 promoter from latency. H12 and 2D12 cells activated with TNF-α and PMA for 24 h (86% EGFP^+^ H12 cells and 28% of EGFP^+^ 2D12 cells) were separated by FACS according to the expression of EGFP and analyzed for CpG methylation. Percentages of methylated CpGs are presented as a mean±SEM.

Next, we investigated directly whether reactivation of latent HIV-1 provirus is accompanied by demethylation of the 5′ LTR. For this purpose, we stimulated H12 and 2D12 cells with TNF-α and PMA for 24 h and performed methylation analysis in sorted EGFP^+^ and EGFP^−^ cells (Figure 3). The proportion of methylated CpG in sorted EGFP^+^ H12 cells and EGFP^+^ 2D12 cells was not significantly changed in comparison to non-sorted H12 and 2D12 cells. The proportion of methylated CpG significantly increased in negatively sorted EGFP^−^ H12 cells (as already shown in Figure 2A). It did not change, however, in sorted EGFP^−^ 2D12 cells, where the HIV-1 promoter is already heavily methylated. Taken together, these results show that reactivation of HIV-1 promoter does not require immediate demethylation.

To show that the differences between H12 and 2D12 cells were not caused by the different capacity to methylate their DNA, we quantified the global CpG methylation level in genomic DNA of H12 and 2D12 cells. The differences between these clones were not statistically significant (71±2% of methylated CpG in H12 cells and 68±6% of methylated CpG in 2D12 cells).

### Differential Susceptibility of Latent HIV-1 with Hypomethylated and Hypermethylated Promoters to Reactivation Signals

**Figure 4:**
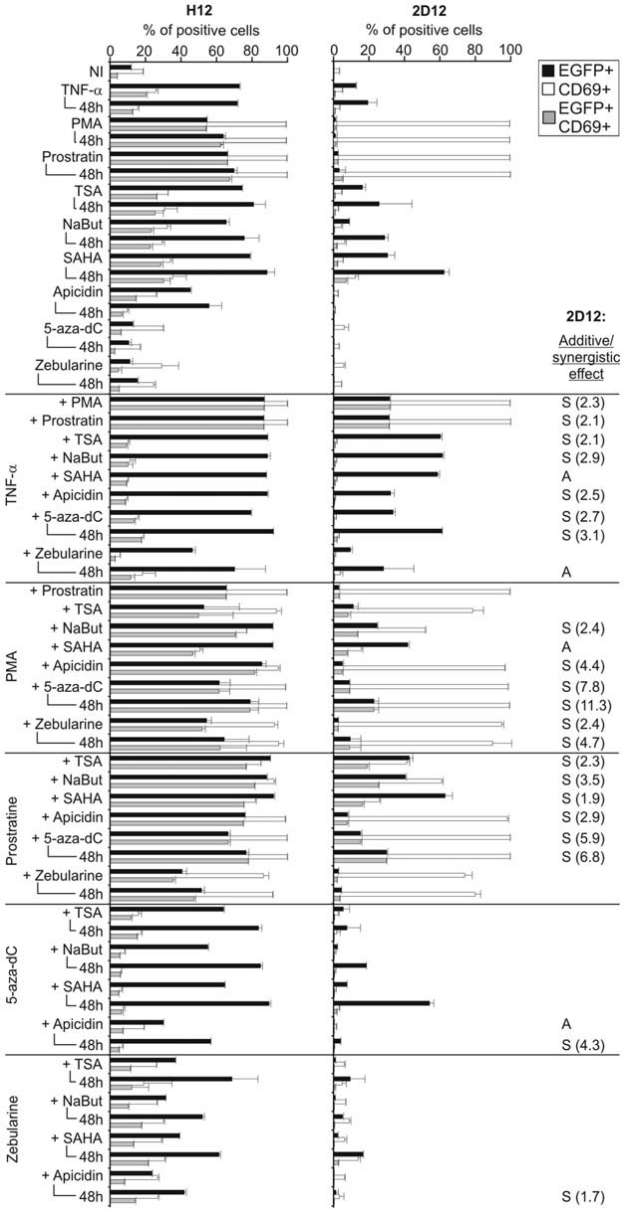
Reactivation of HIV-1 latency in clones harboring proviruses with densely (2D12) and weakly (H12) methylated 5′ LTR. Percentages of EGFP^+^, CD69^+^, and EGFP^+^/CD69^+^ cells in the population of H12 and 2D12 clonal cell lines stimulated with the indicated substances were determined by means of flow cytometry. All data were obtained by gating for live cells after vital staining with Hoechst 33258. Results that represent duplicates or several independent experiments are presented as means±SD. Additive (A) and synergistic (S) effects between two inducers of HIV-1 reactivation are assigned for 2D12 cell line. The index of synergism (in brackets) was determined from the following formula: the percentage of EGFP^+^ cells after stimulation with the combination of stimulators divided by the sum of percentages of EGFP^+^ cells after stimulation with the stimulators separately. As synergistic were considered the combinations resulting in the index of synergism >1.5. As additive were considered the combinations resulting in the index of synergism ≤1.5 and in >30% increase of stimulation in comparison to stimulation with one out of two stimulators.

To assess the resistance of latent HIV-1 with densely methylated promoter to reactivation we exposed both H12 and 2D12 clones to TNF-α, to protein kinase C (PKC) agonists (PMA and prostratin), to inhibitors of HDAC (sodium butyrate (NaBut), trichostatin A (TSA), suberoylanilide hydroxamic acid (SAHA), and apicidin), to inhibitors of DNA methyltransferases, DNMT (5-aza-2-deoxycytidine (5-aza-dC) and zebularine), and/or to their combinations (Figure 4). Whereas cell activation levels as measured by expression of the CD69 marker were similar in both cell lines, reactivation of latent HIV-1 promoter was much stronger in the H12 cell line than in the 2D12 cell line. Reactivation of the HIV-1 LTR was determined after 24-h exposure to reactivation signals. In addition, reactivation with DNMT inhibitors, weak stimulators of the HIV-1 LTR, was determined also after 48-h treatment. To measure reactivation of the HIV-1 LTR on comparable basis, all data were obtained by gating for live cells after vital staining. Among the examined inducers, HDAC inhibitors, including TSA, NaBut, SAHA, and their combinations with other HIV-1-reactivating compounds showed the highest cell toxicity (Figure S2).

The most important relative differences in reactivation of HIV-1 promoter in H12 and 2D12 cells were detected for the agonists of PKC. PMA induced 54% and prostratin 66% of EGFP^+^ H12 cells but failed to activate heavily methylated LTRs in 2D12 cells (as few as 1% and 2% of EGFP^+^ 2D12 cells, respectively). The most efficient inducers of reactivation of the densely methylated HIV-1 promoter in 2D12 cells were inhibitors of HDAC, including TSA, NaBut and SAHA. Importantly, the HDAC inhibitors did not induce significantly higher expression of CD69 than that found in non-induced control cells. Inhibitors of DNMT did not increase the percentages of EGFP^+^ H12 cells over the background value (13% in both zebularine- and 5-aza-dC-treated cultures vs. 12% in non-induced H12 cells), and did not induce significant levels of EGFP in 2D12 cells (0.02% with zebularine and 0.04% with 5-aza-dC). The most efficient combinations of inducers in 2D12 cells were combinations of TNF-α or prostratin with inhibitors of HDAC (TSA, NaBut, and SAHA), and combinations of TNF-α or 5-aza-dC with SAHA. The strongest synergistic effects (synergistic index >4) were observed between PMA and apicidin, 5-aza-dC, or zebularine and between 5-aza-dC and prostratin or apicidin. Although reactivation of HIV-1 from latency in the 2D12 cell line in comparison with the H12 cell line was strongly reduced, most tested compounds induced low but significant expression of the HIV-1 promoter in 2D12 cells.

### Hypermethylated HIV-1 5′ LTR Is Associated with Repressive Chromatin Structure

**Figure 5:**
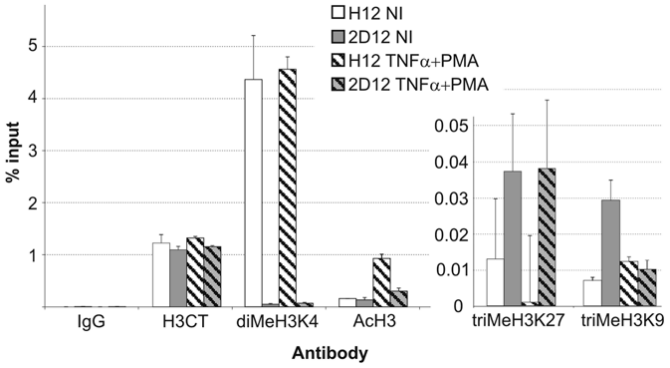
Chromatin structures associated with active and latent states of HIV-1 promoter. Histone 3 (H3) modifications of HIV-1 promoter in the H12 and 2D12 cell lines were analyzed by means of the quantitative ChIP assay using the indicated antibodies before (NI) and after stimulation with TNF-α (10 ng/ml) and PMA (10 nM) (H3CT, anti-H3 C-terminus antibody; diMeH3K4, anti-H3 dimethylated on lysine 4 antibody; AcH3, anti-acetyl-H3 antibody; triMeH3K9, anti-H3 trimethylated on lysine 9 antibody, triMeH3K27, anti-H3 trimethylated on lysine 27 antibody). The equal quantities of HIV promoter immunoprecipitated with anti-H3 C-terminal domain antibody in both cell lines before and after stimulation shows that the total quantity of H3 remained constant. As a control, the cell extracts were immunoprecipitated with normal rabbit IgG. The amount of immunoprecipitated material was normalized to the input DNA. Note that quantities of immunoprecipitated DNA cannot be compared between different H3 modifications, as quantitation is not absolute and depends on antibody affinity. The experiments were performed in triplicates.

To elucidate the contribution of chromatin structure to the maintenance of the latent state of HIV-1, we investigated histone modifications at hypo- and hypermethylated HIV-1 5′ LTRs (Figure 5). We found that the levels of histone 3 (H3) dimethylated on lysine 4 (diMeH3K4), representing active chromatin, that were associated with HIV-1 promoter were significantly lower in 2D12 cells than in H12 cells (89.8 times lower in non-stimulated cells and 65.7 times lower in stimulated cells). In contrast, the levels of acetylated H3, another marker of active chromatin, in non-stimulated cells of both cell lines were similar. After stimulation, the levels of acetylated H3 increased (6.2 times in H12 cells and 2.3 times in 2D12 cells). The levels of H3 trimethylation on lysine 9 (triMeH3K9), representing repressive chromatin, in non-activated cells were 4.1 times higher in cells of the 2D12 line than in cells of the H12 line. The levels of triMeH3K27, also representing repressive chromatin, dropped 13.1 times after activation of H12 cells. In non-activated cells, the triMeH3K27 levels were 2.9 times higher in 2D12 cells than in H12 cells. Whereas after activation the level of H3K9 trimethylation in cells of the H12 line did not significantly change, it decreased 2.9 times in cells of the 2D12 line. Thus, hypermethylated DNA of proviral promoter in 2D12 cells is more strongly associated with repressive chromatin structure than hypomethylated DNA of proviral promoter in H12 cells.

### Hypermethylation of the HIV-1 5′ LTR in Long-Term Plasma Viremia Negative HIV-1-Infected Individuals

**Figure 6:**
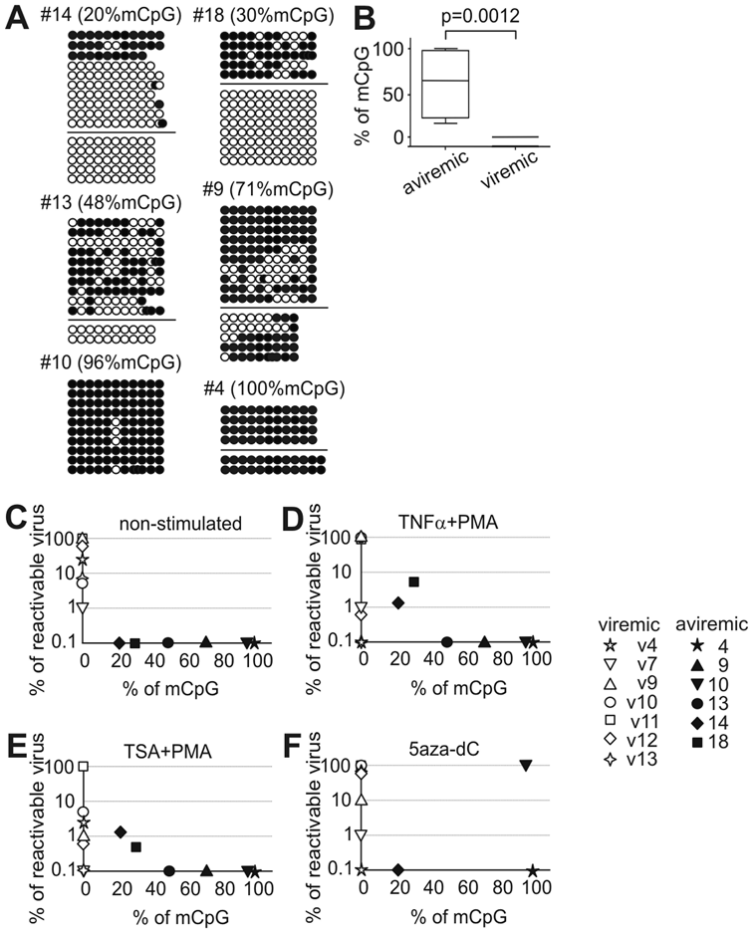
Methylation analysis of HIV-1 promoter in memory CD4^+^ T cells purified from HIV-1-infected individuals. Hypermethylation of the HIV-1 5′ LTR in HIV-1-infected long-term aviremic individuals contrasts with hypomethylation of the HIV-1 5′ LTR in viremic patients and negatively correlates with reactivation of the HIV-1 provirus. (A) CpG methylation patterns in the bisulfite-treated HIV-1 5′ LTR sequences of aviremic patients were clustered using neighbor-joining method into several groups within each patient (separated by horizontal bars) with a strong bootstrap support (>990/1000). The sequences within each group differed in addition in about 1 to 10 point-mutations. Note that the variability of bisulfite-treated sequences is underestimated due to the conversion of the majority of cytosine residues to thymine (except for cytosine in 5-methylcytosine residues). Open circles, nonmethylated CpG residues; closed circles, methylated CpG residues. (B) Percentage of methylated CpGs (mCpG) in the 5′ LTR of HIV-1 in long-term aviremic (n = 6) and viremic (n = 7) patients. *p*, non-parametric, two side Mann-Whitney test. (C–F) Reactivation of HIV-1 provirus in memory CD4^+^ T cells obtained from patients with different levels of CpG methylation. Memory CD4^+^ T cells cultured for three days in the presence of reactivating agents were 10-fold serially diluted in duplicate and co-cultured with PHA-activated CD4^+^ T cells from an allogeneic healthy donor. HIV-1 replication was followed by determination of p24 in the cell-free supernatant. Percentage of reactivable provirus is presented as a ratio of end-point dilutions of patients' CD4^+^ T cells producing HIV-1 virus to the number of DNA proviral copies in the cell quantity equivalent to end-point dilution and determined by quantitative PCR, as indicated in Table 1. (C) Non-stimulated CD4^+^ T cells. (D) CD4^+^ T cells stimulated with TNF-α at 10 ng/ml and 10-nM PMA. (E) CD4^+^ T cells stimulated with 500-nM TSA and 10-nM PMA. (F) CD4^+^ T cells stimulated with 5-µM 5-aza-dC. (C–F) Open symbols, viremic patients; closed symbols, aviremic patients.

Our next aim was to compare the methylation pattern of latent HIV-1 promoter in our *in vitro* model with that in infected individuals. We inspected the 5′ LTR CpG methylation in latent reservoirs of resting CD4^+^ T cells in patients in whom HAART resulted in long-term suppression of plasma viremia and in viremic patients (Table 1 and Table S3). The low percentage of HIV provirus-harboring cells in the latent reservoir and the low sensitivity of PCR after bisulfite treatment of DNA hampered determination of the methylation pattern in several analyzed patients. Thus, from a cohort of 18 HAART-treated patients without detectable plasma viremia, we analyzed six individuals, and from 13 viremic patients we analyzed seven individuals with ≥10^3^ HIV-1 DNA copies per million CD4^+^ cells (Table 1 and Figure 6A and B).

**Table 1:** CpG methylation of HIV-1 promoter and quantitation of truncated HIV-1 in patients without detectable plasma viremia and in viremic controls.

	No.	Age	Sex	Therapy^a^	On therapy from	CD4/mm^3^	Plasma charge (copies/ml)	DNA proviral copies/10^6^ cells^b^	LTR/tat^c^	CpG methylation (%)
Aviremic	4	41	M	2 NRTI+NNRTI	1992	586	<50	2×10^3^	0.5 (ns)	100
	9	43	M	2 NRTI+PI	1996	417	<50	4×10^3^	200 (*p* = 0.002)	74
	10	37	M	2 NRTI+NNRTI	1997	958	<50	1×10^3^	1.5 (ns)	96
	13	47	F	2 NRTI+PI	1995	175	<50	1×10^5^	ND	61
	14	35	F	2 NRTI+PI	2000	650	<50	8×10^3^	0.97 (ns)	19
	18	54	M	2 NRTI+PI	2005	540	<50	2×10^3^	3.7 (ns)	30
Viremic	v4	36	M	TN	-	556	108,843	4×10^3^	ND	0
	v7	67	M	TN	-	638	101,107	1×10^3^	ND	0
	v9	42	M	2 NRTI+PI	1997	580	69,177	1×10^4^	ND	0
	v10	49	M	2 NRTI+PI	1989	144	420	2×10^3^	14 (*p* = 0.01)	0
	v11	57	M	NRTI+NNRTI+PI	1995	661	2,721	5×10^3^	2190 (*p* = 0.001)	0
	v12	50	M	2 NRTI+PI	2008	400	600	1.7×10^4^	13 (*p* = 0.02)	0
	v13	47	F	2 NRTI+PI	1995	200	1,432	1.5×10^4^	ND	0.1

a NRTI, nucleoside reverse transcriptase inhibitor; NNRTI, non-nucleoside reverse transcriptase inhibitor; PI, protease inhibitor; TN, treatment naïve. *See* Table S3 for more details.

b Determined by quantitative PCR as described in Protocol S1.

c Proportion of truncated to full-length proviruses. The titers of LTR and *tat* sequences were determined by quantitative PCR as described in Protocol S1. ns, non-significative difference, *p*, non-parametric, two side Mann-Whitney test; ND, not-determined.

The 5′ LTR of HIV-1 in six patients without detectable plasma viremia contained 20%, 30%, 48%, 71%, 96%, and 100% of methylated CpGs compared with <0.1% of methylated CpGs in HIV-1 promoters in a control group of viremic patients (p = 0.0012, Figure 6B). This striking difference shows that the high level of CpG methylation is a specific characteristic of HIV-1 promoters in aviremic patients. However, numerous non-methylated proviral sequences were found, particularly in two patients with the lowest level of methylated CpGs (patients 14 and 18). We further analyzed the sequence variability of HIV-1 promoters of aviremic patients in the set of sequences obtained after bisulfite treatment. Although rigorous phylogenetic analysis of the bisulfite-treated 5′ LTR of HIV-1 sequences is precluded by the conversion of the majority of cytosine residues to thymine (except for methylated cytosine residues), we used the neighbor-joining method to compare different sequences of HIV-1 promoters. In three aviremic patients harboring the lowest proportion of methylated CpGs in the 5′ LTR of HIV-1 (patients 13, 14, 18), the non-methylated and methylated sequences clearly segregate in different sequence clusters (Figure 6A). Also densely methylated sequences (patients 4 and 9) clustered in two different groups. In conclusion, limited numbers of HIV-1 proviruses with hypermethylated promoters are present in the long-term HAART-treated aviremic individuals in contrast to non-methylated promoters of viremic patients.

### Truncated HIV-1 Sequences Do Not Prevail in Memory CD4^+^ T Cells of Aviremic Patients

Clusters of identical or nearly-identical 5′LTR sequences point to a usually observed variability of HIV-1 sequences in infected persons and arise the question whether the HIV-1 sequences analyzed for the methylation profile represent replication-competent or defective proviruses. To this end, we assayed whether a significant subset of defective proviruses consisting of extremely deleted HIV-1 genomes [41] is more prevalent in memory CD4^+^ T cells of viremic or aviremic patients. This subset of defective proviruses, detected in PBMCs of most HIV-1-infected persons, can be formed by reverse transcriptase jumps over the central part of HIV-1 genomes, so that the resulting molecules more frequently contain the LTRs than the central sequences of HIV-1 genome [41]. Quantitative PCR was used to compare the quantity of the central (*tat* gene) and terminal (LTR) HIV-1 sequences, and to determine the LTR/*tat* ratio (Protocol S1 and Table 1). Therefore, by means of the LTR/*tat* ratio we estimated the proportion of truncated to full-length proviruses in viremic and aviremic patients. We have not found any significant differences between the levels of *tat* and LTR sequences in DNA of aviremic patients 4, 10, 14, and 18. In DNA of all tested viremic patients, we have found prevailing terminal LTRs over *tat* sequences, consistently with our previous demonstration that accumulation of truncated proviruses is a consequence of HIV-1 replication in infected individuals [41],[42]. Thus, truncated proviruses are not prevalent in the majority of aviremic patients.

### Tight Control of HIV-1 Latency *ex vivo* Is Related to Hypermethylation of the HIV-1 5′ LTR of HIV-1-Infected Individuals

Finally, we investigated whether the density of CpG methylation of HIV-1 promoters harbored in memory CD4^+^ T cells of long-term aviremic individuals correlates with the resistance of HIV-1 to reactivation signals. To this end, purified memory CD4^+^ T cells of HIV-1-infected individuals were stimulated with TNF-α and PMA, with TSA and PMA, and with 5-aza-dC for three days; they were serially diluted and co-cultured with allogeneic phytohemagglutinin (PHA)-activated CD4^+^ T cells; and the quantity of HIV-1 secreted to cell-free supernatant was determined (Figure 6C–F). The results show a negative correlation between methylation levels of the HIV-1 5′ LTR and percentage of reactivable proviruses *ex vivo* calculated on the basis of end point titers from aviremic individuals with combinations of TNF-α and PMA and of TSA and PMA (Figure 6D and E). These HIV-1 inducers reactivated proviruses only from the memory CD4^+^ T cells of long-term aviremic individuals harboring high proportions of non-methylated promoters (patients 14 and 18). Neither the combination of TNF-α and PMA nor that of TSA and PMA reactivated HIV-1 proviruses with promoters containing ≥48% of methylated CpGs (n = 4). Although memory CD4^+^ T cells were stimulated in the presence of IL-2 and in the absence of inhibitors of integrase, no outgrowth of possibly non-integrated proviruses was observed in the cells of 4 out of 6 aviremic patients. Reactivation of HIV-1 provirus containing 96% of methylated CpGs (n = 1) with 5 µM 5-aza-dC shows that memory CD4^+^ T cells of this patient contained replication-competent virus, which could be reactivated by the demethylating agent (Figure 6F). In contrast to proviruses in aviremic individuals, which are hardly reactivable *ex vivo*, CD4^+^ T cells of all viremic patients produced HIV-1 as a result of three-day culture in the presence of IL-2 followed by a simple co-culture with allogeneic PHA-activated CD4^+^ T cells (Figure 6C).

## Discussion

Our present work demonstrates that CpG methylation of the HIV-1 5′LTR could be an important epigenetic mechanism that maintains the latency of HIV-1 proviruses by preventing their reactivation. Our results explain the ambiguous conclusions emerging from methylation studies in cell lines latently infected with HIV-1 [31]–[36],[43] and, of particular importance, correlate the *in vitro* data with data from HIV-1-infected patients. CpG methylation in HIV-1-infected patients has not been studied so far. In spite of the low number of HIV provirus-harboring cells in the latent reservoir and the decreased sensitivity of PCR after bisulfite treatment of DNA, our work shows the possibility of studying, and possibly acting upon, the epigenetic control of HIV-1 latency in infected individuals.

Tight control of HIV-1 latency by CpG methylation of the 5′ LTR could be important for the maintenance of, and ultimately for purging, the reservoir of latently infected cells in infected individuals. The high resistance of densely methylated latent HIV-1 proviruses in the 2D12 cell line and in CD4^+^ T cells of aviremic patients to the majority of HIV-1-reactivating compounds tested in our work illustrates the difficulty of purging the reservoir of latent HIV-1 with T-cell-activating agents such as IL-2 and OKT3 [1],[2],[44], prostratin [45]–[47], and IL-7 [47]–[49]. On the other hand, the resistance of densely methylated HIV-1 proviruses to reactivation signals was incomplete, and we can assume that even these proviruses are not stably latent and could produce infectious progeny after suitable stimulation. Thus, a high methylation level of the 5′ LTR contributes to the multiple mechanisms controlling HIV-1 latency that make eradication of HIV-1 problematic, unless new approaches are developed.

In addition to epigenetic mechanisms, the expression of HIV-1 provirus can be abrogated by genetic defects. The 5′LTR sequences from infected viremic or aviremic patients analyzed by bisulfite sequencing did not show any significant mutations in comparison with corresponding sequences present in the HIV-database [50]. It is, however, difficult to screen genetic defects in the inner HIV-1 sequences; long-distance sequence analysis of DNA adjacent to LTR is hampered on the bisulfite-treated template and our analysis of defective viruses detects just gross deletions containing the *tat* region. Using the 5-aza-dC, we have rescued the replication-competent virus from one patient (No. 10) where 5′LTR sequences display almost full methylation of all CpGs. Although we cannot completely exclude the possibility of virus rescue from a non-methylated provirus that escaped our methylation analysis, this result strongly suggests that the parental replication-competent HIV-1 proviruses in this patient contain nearly fully methylated promoters. At the moment, we are unable to quantify the proportion of the latent replication-competent proviruses in the whole proviral population. As the first approximation we used a quantitative assay, by means of which we detected significantly higher proportion of defective truncated proviruses in viremic than in aviremic patients. This testifies against the possibility that methylated proviruses are preferentially defective.

**Figure 7:**
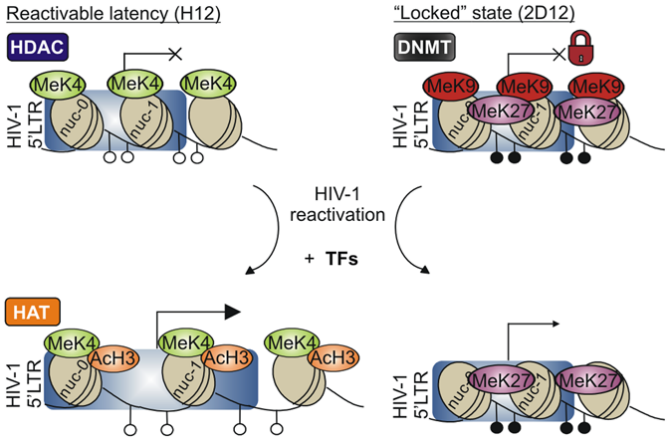
Two-step model of epigenetic control of HIV-1 latency. In the state of reactivable latency exemplified by the H12 cell line, the HIV-1 promoter is hypomethylated, histone 3 (H3) is methylated on lysine 4 (MeK4), but deacetylated by histone deacetylases (HDAC), nuc-0 and nuc-1 are in proximal position; chromatin is condensed. After reactivation with TNF-α and PMA, the hypomethylated HIV-1 promoter-associated H3 is acetylated by means of histone acetyltransferases (HAT). Nucleosomes nuc-0 and nuc-1 are in distal position. In the “locked” silent state exemplified by the 2D12 cell line, the HIV-1 promoter is methylated by DNMT, and H3 is demethylated on K4 and methylated on K9 (MeK9) and K27 (MeK27). After reactivation of the hypermethylated HIV-1 promoter the HIV-1 LTR-associated H3 remains methylated on K27 but is demethylated on K9; CpG methylation is not changed and the repression is overcome by excess of transcription factors. The percentage of cells harboring reactivated HIV-1 is markedly lower in the clone with the “locked” highly methylated promoter (2D12) than in the clone with hypomethylated promoter (H12). Open circles, nonmethylated CpG residues; closed circles, methylated CpG residues, TFs, transcription factors.

We extended here our previous *in vitro* results showing that CpG methylation of the HIV-1 5′ LTR is not necessary for the establishment of viral latency [35]. Indeed, the latent reservoir of some aviremic patients contained high proportions of the non-methylated 5′ LTR. However, the latency controlled solely by transcriptional interference and by chromatin-dependent mechanisms tends to be leaky and easily reactivable. On the basis of our results, we propose a two-step model of epigenetic control of HIV-1 latency (Figure 7). The hypomethylated HIV-1 5′ LTR in the state of reactivable latency in the clone H12 is significantly less associated with acetylated H3 chromatin structure than that in the active state after provirus reactivation. In contrast, dense CpG methylation of the proviral promoter (exemplified by the clone 2D12), probably together with hallmarks of the repressive histone code such as low levels of H3K4 di-methylation and high levels of H3K9 and H3K27 tri-methylation, could contribute to the “locked” silent state of the provirus and make the return of the provirus to an active state inefficient [51].

Reactivation of HIV-1 promoter is not accompanied by significant demethylation of CpG in the 5′ HIV-1 LTR (Figure 3); however, it is accompanied by a rapid drop of H3K9 trimethylation. Indeed, inhibitors of DNMT alone do not induce reactivation of the HIV-1 promoter in 2D12 cells. In contrast, the HDAC inhibitors, TSA, NaBut, and namely SAHA, efficiently reactivate the densely methylated HIV-1 promoter. Moreover, HDAC inhibitors, which are generally anti-inflammatory [52], reactivate HIV-1 latency in virtual absence of T cell activation; this corroborates the results shown previously in the context of HIV-1 reactivation from latency by transcription factor ΔVII-Ets-1 [17]. Activation of CD4^+^ T cells is, therefore, dispensable for reactivation of HIV-1 provirus. Among assayed inducers of reactivation of HIV-1 promoter, only PKC agonists were concomitantly strong inducers of the early marker of activation of T cells, CD69. Thus, chromatin changes precede CpG methylation of the 5′ LTR in the course of establishment of HIV-1 latency, and chromatin changes also initiate reactivation of latent HIV-1 in spite of the presence of demethylated CpG (Figure 7). This kinetics corresponds well with the fact that passive demethylation occurring in the S-phase of the cell cycle is rather slow to precede the rapid onset of activation. In contrast to our results, a clear trend toward demethylation of CpG has been observed after 48 hours of TNF-α treatment of cell line ACH-2 [34]. CpG demethylation after reactivation of latent HIV-1 by different signals in different cell lines is intensively studied in our laboratories.

As in the majority of latently infected cells in infected individuals [53],[54], the HIV-1 provirus in the H12 cell line is integrated within a gene. Surprisingly, distinct levels of resistance of HIV-1 provirus to reactivation could be established at the same integration site. Thus, the HIV-1 latency in H12 cells is reactivation-prone, whereas in 2D12 cells with the provirus inserted at exactly the same genomic site the HIV-1 latency is reactivation-resistant. Both distinct states of HIV-1 latency are quasi-stable during cell divisions and both states are re-established after the return of reactivated HIV-1 to a basal latent state. Positive selection of EGFP^+^ H12 cells harboring reactivated virus also results in the return to a basal latent state with an unchanged methylation profile in the absence of the activation stimulus; however, tightly controlled HIV-1 proviruses could be negatively selected from the population of EGFP^−^ H12 cells resistant to HIV-1-reactivating compounds. At the moment, we study frequency of highly restricted viruses negatively selected from additional clones harboring HIV-1 proviruses with hypomethylated promoters.

HIV-1 latency is compatible with various DNA methylation levels. We suppose that both extremes of methylation density can be selected in different experimental settings as a function of reactivation-proneness of latently infected cells. During the development of models of reactivable latency [37]–[40], clones harboring readily reactivable provirus have been selected from the population of cells following integration of the virus in the absence of viral gene expression (silent integration) [37]. As exemplified by the cell line H12, selection of such cell lines results in proliferation of cell clones with hypomethylated 5′ LTR and leaky control of provirus transcription. In inverse order to the events performed in previous works [37]–[40], we negatively selected from the population of cell line H12 the cells harboring a latent provirus resistant to reactivation. This procedure resulted in the outgrowth of cell clones with hypermethylated 5′ LTR and tight control of provirus transcription, exemplified by the cell line 2D12. It is possible that a similarly tight control of HIV-1 promoter reactivation by hypermethylation was necessary to select latently infected cells by silencing of actively replicating virus from chronically infected cell lines U937 and ACH-2 [55]–[57]. Indeed, HIV-1 promoter in these historically first described latent cell lines is heavily methylated [33],[34].

It is difficult to speculate about the exact transcriptional status of heavily methylated HIV promoters in aviremic patients and their contribution to residual viremia [58]–[64]. Their reactivation is probably a rare and stochastic event as suggested by the sequential homogeneity of virus rebounding during the short structured treatment interruptions [58]. While immunologic stimuli *in vivo* are adequate to overcome CpG methylation-mediated suppression of HIV-1 expression in virtually all patients who discontinued HAART, resistance of highly methylated HIV-1 promoters in memory CD4^+^ T cells purified from aviremic patients to reactivation *ex vivo* could be related to low HIV-1 DNA copy numbers in assayed aliquots. Our *ex vivo* experiments suggest that proviruses with densely methylated promoters coexist in some aviremic patients with latent but easily reactivable proviruses controlled by non-methylated promoters. In contrast to aviremic patients, non-methylated promoters present in viremic patients are expressed in the absence of external stimuli. HIV-1 was rescued from virtually all provirus-containing cells of viremic patients, without any evidence of DNA methylation. The non-methylated status of HIV-1 promoters in viremic patients might be further enhanced by the presence of unintegrated viral DNA, abundant during the viremic phase of HIV-1 infection [65]. Thus, the virus recovered from memory CD4^+^ T cells of viremic patients may represent, in addition to the product of integrated proviral genomes, also the product of pre-integrated HIV-1 DNA.

We suppose that during the acute phase of HIV-1 infection accompanied by stimulation of the immune system [66]–[70], memory CD4^+^ T cells harboring reactivation-resistant latent HIV-1 that contained hypermethylated promoters have a better chance to escape from virus-induced cytopathic effects and cytotoxic responses than those infected with reactivation-prone hypomethylated provirus. Thus, the presence of hypermethylated HIV-1 promoters in CD4^+^ T cells of patients with long-term suppressed viremia could be the result of selection of proviruses with tightly controlled latency. In contrast, memory CD4^+^ T cells harboring reactivation-prone HIV-1 proviruses with hypomethylated promoters could be counter-selected by a virus-specific cytopathic effect and cytotoxic responses during perpetual stimulation of the immune system. Patients' cells analyzed in our experiments were sorted for memory, but not activation, CD4^+^ T phenotype. It is probable that the frequency of activated memory cells is lower in aviremic than in viremic patients and that the difference in the methylation pattern between both groups is strengthened by this effect.

The multitude of mechanisms that control HIV-1 latency is a cause of our inability to purge the reservoir of latent HIV-1 in infected individuals. Methylation of the HIV-1 5′ LTR is an additional restriction mechanism, which controls resistance of latent HIV-1 against reactivation signals and thus determines the stability of the HIV-1 latency, although it is not required for the initial silencing events and it needs not to be reverted immediately during reactivation. Reactivation of HIV-1 latency in virtual absence of T cell activation by SAHA, an HDAC inhibitor already approved for use in humans, and strong synergistic effects between 5′aza-dC and other activators of HIV-1, may represent an important step toward the elimination of the latent HIV-1 reservoir. CpG methylation accompanied by repressive histone code seems to be the ultimate step in the development of the “locked” silent state of the provirus. The high stability of the locked silent state is, however, not complete. High but incomplete resistance of HIV-1 to reactivation represents a therapeutic challenge for the future.

## Materials and Methods

### Ethics Statement

This study was conducted according to the principles expressed in the Declaration of Helsinki. Each patient provided informed written consent to participation in this study in accordance with institutional and regulatory guidelines. The study was approved by the Institutional Review Board of Ambroise Paré Hospital.

### Patients

Seropositive individuals were selected for the study on the basis of at least two years of lasting infection and non-detectable plasma viral load during the last year (<50 HIV-1 RNA copies per milliliter of plasma as determined with Amplicore Ultrasensitive HIV-1 Monitor Assay; Roche Molecular Diagnostic System). Viremic individuals with plasma viremia from 6×10^4^ to 1×10^5^ RNA copies per milliliter of plasma were analyzed as controls.

### Preparation of Memory CD4^+^ T Cells from PBMCs of HIV-1-Infected Patients

Patients' PBMCs (aliquots of 2×10^7^ cells) were separated on Ficoll-Hypaque gradients. Memory CD4^+^ T cells separated from PBMC by means of a memory CD4^+^ T cell isolation kit (Miltenyi Biotech, France) were cultured at the concentration of 10^6^ cells per milliliter of RPMI 1640 supplemented with 200-U/ml recombinant IL-2 (Chiron), 15% fetal calf serum, and antibiotics in duplicate in a total volume of 100 µl in the presence of HIV-1-reactivating agents. After a 3-day stimulation, CD4^+^ T cells were 10-fold serially diluted and co-cultured with PHA-activated CD4^+^ T cells from an allogeneic healthy donor, as described in detail previously [71]. HIV-1 replication was followed by determination of p24 in the cell-free supernatant by means of Genetic System HIV Ag EIA (BioRad France, Marnes la Coquettes). CD4^+^ T cells infected with HIV-1 NL4-3 or with HIV-1 AD8 were used as positive controls.

### Clonal Cell Lines

Cell lines A2, A8, G10, and H12 were prepared by transduction of Jurkat cells with the HIV-1-based vector (pEV731 vector from the pHR' series) containing Tat and EGFP under the control of the HIV-1 LTR (LTR-Tat-IRES-EGFP-LTR), as described previously [38]. The JNLGFP Jurkat cell line contains multiple copies of integrated full-length HIV-1 provirus including the EGFP open reading frame placed between the *env* and *nef* genes [40]. H12 cells stimulated with TNF-α and PMA were separated by cell sorting of EGFP negative cells with FACSAria (BD Biosciences).

### Reactivation of Latent HIV-1 Provirus

To reactivate the HIV-1 provirus from latency, we treated the cell lines or patients' memory CD4^+^ T cells with 10-nM PMA (Sigma-Aldrich), TNF-α (Sigma-Aldrich) at 10 ng/ml, 1 or 5-µM 5-aza-dC (Sigma-Aldrich), 500-nM TSA (Sigma-Aldrich), 5-µM prostratin, 5-mM NaBut, 2.5-µM SAHA, 0.5-µM apicidin and 500-µM zebularine, or with their different combinations.

### Flow Cytometry Analysis

We analyzed EGFP^+^ and anti-CD69-phycoerythrin-labeled (Becton-Dickinson) viable cells, negative for staining with Hoechst 33258 (Sigma-Aldrich) at 1 µg/ml, with LSR II and FACSCalibur flow cytometers, using CELLQuest and FlowJo software (Becton-Dickinson, Le Pont de Claix, France). EGFP+ cells were isolated using FACSAria cell sorter (Becton Dickinson).

### Cloning of H12 Cells

Aliquots, 200 µl each, of H12 cells diluted to the concentration of one cell per 2 milliliters were distributed into 96-well microtiter plates as specified in Protocol S1. Subcultures were expanded further.

### Bisulfite Cytosine Methylation Analysis

The samples of total genomic DNA isolated with the QIAamp DNA Blood Mini Kit (Qiagen Inc.) were digested overnight with *Eco*RI and prepared for methylation analysis as described by Hajkova et al. [72], or proceeded to methylation analysis by means of the Epitect Bisufite Kit (Qiagen Inc.).

Bisulfite-treated DNA was amplified by means of PCR in a 50-µl reaction mixture containing 50 mM Tris-HCl (pH 9.2), 1.75 mM MgCl_2_, each dNTP at 350 µM, and each primer at 45 pmol: MB (5′-TGGTAGAATTATATATTAGGGTTAGGGATT-3′, nucleotides [nt] 81 to 110, sense), MH (5′-CACCCATCTCTCTCCTTCTAACCTC-3′, nt 772 to 796, antisense) and MC (5′-AGAGAAGGTAGAAGAAGTTAATGAAGGAGA-3′, nt 161 to 190, sense); MG (5′-AAAAAACTCCTCTAATTTCCCTTT-3′, nt 663 to 686, antisense) or MF instead of MG (5′- AAATCTAAAAAATCTCTAATTACCAAAATC-3′, nt 577 to 606, antisense) for the HIV 5′ LTR. The sense primers contained T and the antisense primers A instead of C in positions complementary to non-methylable C (i.e., C out of CpG dinucleotides). PCR was performed with about 50 ng of genomic DNA for 40 cycles at 95°C for 60 s, 58°C for 120 s, and 72°C for 60 s. Amplification products were cloned in the pGEM-T-Easy Vector System (Promega, Madison, WI) and sequenced. Only PCR clones with at least 95% conversion of cytosines outside CpGs were taken into account.

### Clustering of Patients' HIV-1 LTR Sequences

The sequences of different HIV-1 LTRs amplified after bisulfite treatment of aviremic patients' DNA samples were clustered within each patient using the neighbor-joining method. The bootstrap support was very strong for the distinct clusters. All CpGs were masked for this analysis to exclude the differences in the methylation status.

### Quantification of Global DNA Methylation

The Methylamp™ Global DNA Methylation Quantification Ultra Kit (Epigentek) was used according to the manufacturer's instructions to quantify global DNA methylation of genomic DNA in H12 and 2D12 Jurkat clonal cell lines.

### ChIP

ChIP assays were performed by means of the ChIP assay kit (Upstate Biochemicals, Temecula, CA). H12 and 2D12 Jurkat clonal cell lines were non-induced or induced with TNF-α (10 ng/ml) and PMA (10 nM) 20 min before ChIP assays. The analyzed cells were then cross-linked in the presence of 1% formaldehyde for 10 min at room temperature. Chromatin was sheared by sonication for 45 min in total (30 s ON, 30 s OFF) to obtain DNA fragments of about 200 bp in a Bioruptor (Diagenode SA, Liege, Belgium). The antibodies used for ChIP were as follows: anti-histone H3, CT, pan (Millipore-Upstate, Holsheim, France), anti-acetyl-histone H3 (Millipore-Upstate), anti-dimethyl-histone H3 (Lys4) (Millipore-Upstate), anti-trimethyl-histone H3 (Lys9) (Millipore-Upstate) and anti-trimethyl-histone H3 (Lys 27) (Abcam, Cambridge, UK). For the control, we immunoprecipitated the cell extracts using normal rabbit IgG (Millipore-Upstate) instead of hyperimmune antibody. Immunoprecipitated DNA was subjected to real-time PCR quantification using primers specific for the 5′ LTR (forward: 5′-TTAGCAATGTGTATGGGAGTTGA-3′, reverse: 5′-ATATCTTGTCTTCGTTGGGAGTG-3′).

## Supporting Information

Protocol S1 Quantitative PCR and RT-PCR. Quantification of truncated HIV-1 proviruses. Inverse PCR. Cell cloning.
(0.07 MB PDF)

Table S1 Number of proviral copies in latently transduced Jurkat clonal cell lines.
(0.03 MB PDF)

Table S2 Flanking sequences of HIV-1-based vector integrated in the first intron of the *ubiquilin* gene in the cell line H12.
(0.03 MB PDF)

Table S3 Antiretroviral therapy in patients without detectable plasma viremia (<50 copies/ml).
(0.05 MB PDF)

Figure S1 Selection of H12 cells resistant to reactivation of HIV-1 provirus from latency. (A) Flow chart protocol showing activations of H12 cells with TNF-α and PMA, the first and second cell sorting of EGFP-negative cells, and cultivation periods in the absence of exogeneous activator. (B) Immunofluorescence of EGFP in non-activated H12 Jurkat cells. (C) Immunofluorescence of EGFP in H12 cells exposed to TNF-α (10 ng/ml) and PMA (10 nM) for 24 h. (D) CpG methylation profile of the 5′ LTR in H12 cells exposed to TNF-α and PMA for 24 h and negatively sorted for EGFP. (E) Immunofluorescence of EGFP in H12 Jurkat cells activated with TNF-α and PMA for 24 h, negatively sorted for EGFP, and then kept in culture in the absence of exogeneous activator for 24 days. (F) Immunofluorescence of EGFP in H12 Jurkat cells cultured as in (E) and stimulated for the second time with TNF-α and PMA for 24 h. (G) CpG methylation profile of the 5′ LTR in H12 cells cultured as in (F), negatively sorted for the second time for EGFP. (H) Immunofluorescence of EGFP in H12 Jurkat cells cultured as in (F), negatively sorted for EGFP, and then kept in culture in the absence of exogeneous activator for 60 days. (I) Immunofluorescence of EGFP in H12 Jurkat cells cultured as in (H) and stimulated for the third time with TNF-α and PMA for 24 h. (J) CpG methylation profile of the 5′ LTR in H12 cells cultured as in (H). Open circles, nonmethylated CpG residues; closed circles, methylated CpG residues. Bars in B, C, E, F, H, and I represent percentages of EGFP-positive cells.
(0.13 MB PDF)

Figure S2 Survival of H12 and 2D12 cells treated with HIV-1 inducers. Percentages of live H12 and 2D12 cells relative to non-induced (NI) control were determined by means of vital staining with Hoechst 33258 and flow cytometry after 24 h and/or 48 h stimulation with the indicated substances. Results that represent duplicates or several independent experiments are presented as means±SD.
(0.16 MB PDF)

## References

[1] Chun TW, Engel D, Mizell SB, Hallahan CW, Fischette M (1999). Effect of interleukin-2 on the pool of latently infected, resting CD4+ T cells in HIV-1-infected patients receiving highly active anti-retroviral therapy.. Nat Med.

[2] Davey RT, Bhat N, Yoder C, Chun TW, Metcalf JA (1999). HIV-1 and T cell dynamics after interruption of highly active antiretroviral therapy (HAART) in patients with a history of sustained viral suppression.. Proc Natl Acad Sci U S A.

[3] Strain MC, Gunthard HF, Havlir DV, Ignacio CC, Smith DM (2003). Heterogeneous clearance rates of long-lived lymphocytes infected with HIV: intrinsic stability predicts lifelong persistence.. Proc Natl Acad Sci U S A.

[4] Ramratnam B, Mittler JE, Zhang L, Boden D, Hurley A (2000). The decay of the latent reservoir of replication-competent HIV-1 is inversely correlated with the extent of residual viral replication during prolonged anti-retroviral therapy.. Nat Med.

[5] Finzi D, Blankson J, Siliciano JD, Margolick JB, Chadwick K (1999). Latent infection of CD4+ T cells provides a mechanism for lifelong persistence of HIV-1, even in patients on effective combination therapy.. Nat Med.

[6] Kinoshita S, Chen BK, Kaneshima H, Nolan GP (1998). Host control of HIV-1 parasitism in T cells by the nuclear factor of activated T cells.. Cell.

[7] Nabel G, Baltimore D (1987). An inducible transcription factor activates expression of human immunodeficiency virus in T cells.. Nature.

[8] Tyagi M, Karn J (2007). CBF-1 promotes transcriptional silencing during the establishment of HIV-1 latency.. Embo J.

[9] He G, Margolis DM (2002). Counterregulation of chromatin deacetylation and histone deacetylase occupancy at the integrated promoter of human immunodeficiency virus type 1 (HIV-1) by the HIV-1 repressor YY1 and HIV-1 activator Tat.. Mol Cell Biol.

[10] Kiernan RE, Vanhulle C, Schiltz L, Adam E, Xiao H (1999). HIV-1 tat transcriptional activity is regulated by acetylation.. Embo J.

[11] Ghose R, Liou LY, Herrmann CH, Rice AP (2001). Induction of TAK (cyclin T1/P-TEFb) in purified resting CD4(+) T lymphocytes by combination of cytokines.. J Virol.

[12] Lin X, Irwin D, Kanazawa S, Huang L, Romeo J (2003). Transcriptional profiles of latent human immunodeficiency virus in infected individuals: effects of Tat on the host and reservoir.. J Virol.

[13] Lassen KG, Bailey JR, Siliciano RF (2004). Analysis of human immunodeficiency virus type 1 transcriptional elongation in resting CD4+ T cells in vivo.. J Virol.

[14] Williams SA, Chen LF, Kwon H, Ruiz-Jarabo CM, Verdin E (2006). NF-kappaB p50 promotes HIV latency through HDAC recruitment and repression of transcriptional initiation.. Embo J.

[15] du Chene I, Basyuk E, Lin YL, Triboulet R, Knezevich A (2007). Suv39H1 and HP1gamma are responsible for chromatin-mediated HIV-1 transcriptional silencing and post-integration latency.. Embo J.

[16] Marban C, Suzanne S, Dequiedt F, de Walque S, Redel L (2007). Recruitment of chromatin-modifying enzymes by CTIP2 promotes HIV-1 transcriptional silencing.. Embo J.

[17] Yang HC, Shen L, Siliciano RF, Pomerantz JL (2009). Isolation of a cellular factor that can reactivate latent HIV-1 without T cell activation.. Proc Natl Acad Sci U S A.

[18] Bosque A, Planelles V (2009). Induction of HIV-1 latency and reactivation in primary memory CD4+ T cells.. Blood.

[19] Mahmoudi T, Parra M, Vries RG, Kauder SE, Verrijzer CP (2006). The SWI/SNF chromatin-remodeling complex is a cofactor for Tat transactivation of the HIV promoter.. J Biol Chem.

[20] Treand C, du Chene I, Bres V, Kiernan R, Benarous R (2006). Requirement for SWI/SNF chromatin-remodeling complex in Tat-mediated activation of the HIV-1 promoter.. Embo J.

[21] Gerritsen ME, Williams AJ, Neish AS, Moore S, Shi Y (1997). CREB-binding protein/p300 are transcriptional coactivators of p65.. Proc Natl Acad Sci U S A.

[22] Van Lint C, Emiliani S, Ott M, Verdin E (1996). Transcriptional activation and chromatin remodeling of the HIV-1 promoter in response to histone acetylation.. Embo J.

[23] Koiwa T, Hamano-Usami A, Ishida T, Okayama A, Yamaguchi K (2002). 5′-long terminal repeat-selective CpG methylation of latent human T-cell leukemia virus type 1 provirus in vitro and in vivo.. J Virol.

[24] Taniguchi Y, Nosaka K, Yasunaga J, Maeda M, Mueller N (2005). Silencing of human T-cell leukemia virus type I gene transcription by epigenetic mechanisms.. Retrovirology.

[25] Harbers K, Schnieke A, Stuhlmann H, Jahner D, Jaenisch R (1981). DNA methylation and gene expression: endogenous retroviral genome becomes infectious after molecular cloning.. Proc Natl Acad Sci U S A.

[26] Robbins PB, Skelton DC, Yu XJ, Halene S, Leonard EH (1998). Consistent, persistent expression from modified retroviral vectors in murine hematopoietic stem cells.. Proc Natl Acad Sci U S A.

[27] Hejnar J, Plachy J, Geryk J, Machon O, Trejbalova K (1999). Inhibition of the Rous sarcoma virus long terminal repeat-driven transcription by in vitro methylation: different sensitivity in permissive chicken cells versus mammalian cells.. Virology.

[28] Hejnar J, Hajkova P, Plachy J, Elleder D, Stepanets V (2001). CpG island protects Rous sarcoma virus-derived vectors integrated into nonpermissive cells from DNA methylation and transcriptional suppression.. Proc Natl Acad Sci U S A.

[29] Lavie L, Kitova M, Maldener E, Meese E, Mayer J (2005). CpG methylation directly regulates transcriptional activity of the human endogenous retrovirus family HERV-K(HML-2).. J Virol.

[30] Matouskova M, Blazkova J, Pajer P, Pavlicek A, Hejnar J (2006). CpG methylation suppresses transcriptional activity of human syncytin-1 in non-placental tissues.. Exp Cell Res.

[31] Bednarik DP, Cook JA, Pitha PM (1990). Inactivation of the HIV LTR by DNA CpG methylation: evidence for a role in latency.. Embo J.

[32] Schulze-Forster K, Gotz F, Wagner H, Kroger H, Simon D (1990). Transcription of HIV1 is inhibited by DNA methylation.. Biochem Biophys Res Commun.

[33] Singh MK, Pauza CD (1992). Extrachromosomal human immunodeficiency virus type 1 sequences are methylated in latently infected U937 cells.. Virology.

[34] Ishida T, Hamano A, Koiwa T, Watanabe T (2006). 5′ long terminal repeat (LTR)-selective methylation of latently infected HIV-1 provirus that is demethylated by reactivation signals.. Retrovirology.

[35] Pion M, Jordan A, Biancotto A, Dequiedt F, Gondois-Rey F (2003). Transcriptional Suppression of In Vitro-Integrated Human Immunodeficiency Virus Type 1 Does Not Correlate with Proviral DNA Methylation.. J Virol.

[36] Pannell D, Osborne CS, Yao S, Sukonnik T, Pasceri P (2000). Retrovirus vector silencing is de novo methylase independent and marked by a repressive histone code.. Embo J.

[37] Duverger A, Jones J, May J, Bibollet-Ruche F, Wagner FA (2009). Determinants of the establishment of human immunodeficiency virus type 1 latency.. J Virol.

[38] Jordan A, Defechereux P, Verdin E (2001). The site of HIV-1 integration in the human genome determines basal transcriptional activity and response to Tat transactivation.. Embo J.

[39] Jordan A, Bisgrove D, Verdin E (2003). HIV reproducibly establishes a latent infection after acute infection of T cells in vitro.. Embo J.

[40] Kutsch O, Benveniste EN, Shaw GM, Levy DN (2002). Direct and quantitative single-cell analysis of human immunodeficiency virus type 1 reactivation from latency.. J Virol.

[41] Sanchez G, Xu X, Chermann JC, Hirsch I (1997). Accumulation of defective viral genomes in peripheral blood mononuclear cells of human immunodeficiency virus type 1-infected individuals.. J Virol.

[42] Pion M, Sanchez G, Liska V, Bettendroffer L, Candotti D (2003). Truncated forms of human and simian immunodeficiency virus in infected individuals and rhesus macaques are unique or rare quasispecies.. Virology.

[43] Miyoshi H, Smith KA, Mosier DE, Verma IM, Torbett BE (1999). Transduction of human CD34+ cells that mediate long-term engraftment of NOD/SCID mice by HIV vectors.. Science.

[44] van Praag RM, Prins JM, Roos MT, Schellekens PT, Ten Berge IJ (2001). OKT3 and IL-2 treatment for purging of the latent HIV-1 reservoir in vivo results in selective long-lasting CD4+ T cell depletion.. J Clin Immunol.

[45] Kulkosky J, Culnan DM, Roman J, Dornadula G, Schnell M (2001). Prostratin: activation of latent HIV-1 expression suggests a potential inductive adjuvant therapy for HAART.. Blood.

[46] Biancotto A, Grivel JC, Gondois-Rey F, Bettendroffer L, Vigne R (2004). Dual role of prostratin in inhibition of infection and reactivation of human immunodeficiency virus from latency in primary blood lymphocytes and lymphoid tissue.. J Virol.

[47] Brooks DG, Hamer DH, Arlen PA, Gao L, Bristol G (2003). Molecular characterization, reactivation, and depletion of latent HIV.. Immunity.

[48] Wang FX, Xu Y, Sullivan J, Souder E, Argyris EG (2005). IL-7 is a potent and proviral strain-specific inducer of latent HIV-1 cellular reservoirs of infected individuals on virally suppressive HAART.. J Clin Invest.

[49] Ducrey-Rundquist O, Guyader M, Trono D (2002). Modalities of interleukin-7-induced human immunodeficiency virus permissiveness in quiescent T lymphocytes.. J Virol.

[50] Leitner T, Foley B, Hahn B, Marx P, McCutchan F (2005).

[51] Mutskov V, Felsenfeld G (2004). Silencing of transgene transcription precedes methylation of promoter DNA and histone H3 lysine 9.. Embo J.

[52] Blanchard F, Chipoy C (2005). Histone deacetylase inhibitors: new drugs for the treatment of inflammatory diseases?. Drug Discov Today.

[53] Han Y, Lassen K, Monie D, Sedaghat AR, Shimoji S (2004). Resting CD4+ T cells from human immunodeficiency virus type 1 (HIV-1)-infected individuals carry integrated HIV-1 genomes within actively transcribed host genes.. J Virol.

[54] Lewinski MK, Bisgrove D, Shinn P, Chen H, Hoffmann C (2005). Genome-wide analysis of chromosomal features repressing human immunodeficiency virus transcription.. J Virol.

[55] Folks TM, Justement J, Kinter A, Schnittman S, Orenstein J (1988). Characterization of a promonocyte clone chronically infected with HIV and inducible by 13-phorbol-12-myristate acetate.. J Immunol.

[56] Clouse KA, Powell D, Washington I, Poli G, Strebel K (1989). Monokine regulation of human immunodeficiency virus-1 expression in a chronically infected human T cell clone.. J Immunol.

[57] Folks T, Kelly J, Benn S, Kinter A, Justement J (1986). Susceptibility of normal human lymphocytes to infection with HTLV-III/LAV.. J Immunol.

[58] Joos B, Fischer M, Kuster H, Pillai SK, Wong JK (2008). HIV rebounds from latently infected cells, rather than from continuing low-level replication.. Proc Natl Acad Sci U S A.

[59] Finzi D, Hermankova M, Pierson T, Carruth LM, Buck C (1997). Identification of a reservoir for HIV-1 in patients on highly active antiretroviral therapy.. Science.

[60] Wong JK, Hezareh M, Gunthard HF, Havlir DV, Ignacio CC (1997). Recovery of replication-competent HIV despite prolonged suppression of plasma viremia.. Science.

[61] Bailey JR, Sedaghat AR, Kieffer T, Brennan T, Lee PK (2006). Residual human immunodeficiency virus type 1 viremia in some patients on antiretroviral therapy is dominated by a small number of invariant clones rarely found in circulating CD4+ T cells.. J Virol.

[62] Palmer S, Maldarelli F, Wiegand A, Bernstein B, Hanna GJ (2008). Low-level viremia persists for at least 7 years in patients on suppressive antiretroviral therapy.. Proc Natl Acad Sci U S A.

[63] Imamichi H, Crandall KA, Natarajan V, Jiang MK, Dewar RL (2001). Human immunodeficiency virus type 1 quasi species that rebound after discontinuation of highly active antiretroviral therapy are similar to the viral quasi species present before initiation of therapy.. J Infect Dis.

[64] Chun TW, Justement JS, Lempicki RA, Yang J, Dennis G (2003). Gene expression and viral prodution in latently infected, resting CD4+ T cells in viremic versus aviremic HIV-infected individuals.. Proc Natl Acad Sci U S A.

[65] Sharkey ME, Teo I, Greenough T, Sharova N, Luzuriaga K (2000). Persistence of episomal HIV-1 infection intermediates in patients on highly active anti-retroviral therapy.. Nat Med.

[66] Mattapallil JJ, Douek DC, Hill B, Nishimura Y, Martin M (2005). Massive infection and loss of memory CD4+ T cells in multiple tissues during acute SIV infection.. Nature.

[67] Brenchley JM, Price DA, Douek DC (2006). HIV disease: fallout from a mucosal catastrophe?. Nat Immunol.

[68] Brenchley JM, Ruff LE, Casazza JP, Koup RA, Price DA (2006). Preferential infection shortens the life span of human immunodeficiency virus-specific CD4+ T cells in vivo.. J Virol.

[69] Li Q, Duan L, Estes JD, Ma ZM, Rourke T (2005). Peak SIV replication in resting memory CD4+ T cells depletes gut lamina propria CD4+ T cells.. Nature.

[70] Douek DC, Roederer M, Koup RA (2008). Emerging Concepts in the Immunopathogenesis of AIDS.. Annu Rev Med.

[71] Gondois-Rey F, Biancotto A, Fernandez MA, Bettendroffer L, Blazkova J (2006). R5 variants of human immunodeficiency virus type 1 preferentially infect CD62L- CD4+ T cells and are potentially resistant to nucleoside reverse transcriptase inhibitors.. J Virol.

[72] Hajkova P, el-Maarri O, Engemann S, Oswald J, Olek A (2002). DNA-methylation analysis by the bisulfite-assisted genomic sequencing method.. Methods Mol Biol.

