# Supplementary material for: CpG Methylation Controls Reactivation of HIV from Latency
Source: PLoS Pathog. 2009 Aug 21;5(8):e1000554. doi: 10.1371/journal.ppat.1000554 (PMC2722084; doi:10.1371/journal.ppat.1000554)
Supplement: Protocol S1 — Quantitative PCR and RT-PCR. Quantification of truncated HIV-1 proviruses. Inverse PCR. Cell cloning. (0.07 MB PDF) [file ppat.1000554.s001.pdf]

SUPPLEMENTAL PROTOCOLS

Quantitative PCR

Quantification of HIV-1 copy-numbers in genomic DNA. Total high-molecular-weight genomic DNA was isolated from patients‘ PBMCs, from the H12 clonal cell line, and from the 8E5 cell line, as described elsewhere. The 8E5 cell line is known to contain one copy of the HIV-1 genome per cell. Twofold serial dilutions of 8E5 DNA ranging from 500 to 4,000 cellular genomes per tube were used for construction of the calibration curve. Twofold serial dilutions of the plasmid pNL4-3, containing the whole HIV-1 genome ranging from 500 to 10,000 viral genomes per tube, were used for construction of the plasmid calibration curve. Twofold serial dilutions of genomic DNA of A2, A8, G10, H12, or JNLGFP ranging from 500 to 4,000 cellular genomes per tube were used in the reactions for determination of the proviral copy number. Dilutions of the plasmid containing the fragment of the actin gene, ranging from 500 to 100,000 actin genes per tube, were used for construction of the actin calibration curve. The number of actin genes within the genomic DNA was used as an internal standard. We performed the reactions in triplicates in a total volume of 30 µl using Taqman 2 x master mix and primers specific for the U5 (forward primer; 5'TGTGTGCCCGTCTGTTGTGT3') and the leader (reverse primer; 5’GAGTCCTGCGTCGAGAGATC3’) of the HIV genome. Specific probe (5’FAM-CAGTGGCGCCCGAACAGGGA-TAMRA3’) for the LTR-leader boundary was used. The amplification was done for 40 cycles.

Quantification of truncated HIV-1 proviruses. The relative quantity of truncated proviruses was assessed from the ratio of concentrations of HIV-1 sequences from the central and terminal parts of the HIV-1 genome in 40 to 100 µg of memory $CD4^+$ T

lymphocyte DNA. PCR was performed with the MESA GREEN qPCR MasterMix Plus for SYBR Assay Kit (Eurogentec). The U3 region of LTR was quantified with LTR4/LTR5 primers (LTR4 forward primer 5' GATTGGCAGAACTACACACCAG 3'; LTR5 reverse primer 5' CCAGCGGAAAGTCCCTTGTAG 3'). *tat* sequences were quantified with TatB primers (TatsnB forward primer 5' TTGGGTGTCGACATAGCAGAATAG 3'; TatasB reverse primer 5' TGAGGAGGTCTTCGTCGCTGT 3'). RNA polymerase II gene within genomic DNA was used as an internal standard. Amplification was done for 45 cycles, reactions were performed in triplicates. Results were normalized to amplification efficiency according to the primer pair used and to two LTR copies versus one Tat copy within the pNL4-3 provirus.

Quantitative RT-PCR. We isolated total RNA from H12 and 2D12 clones using the mirVana miRNA Isolation Kit (Ambion) according to manufacturer's instructions. Two sets of total RNA prepared independently were used in quantitative RT-PCR reactions. One microgram of total RNA was used for the reverse transcription in a total volume of 50 μl, with M-MLV Reverse Transcriptase (Gibco) and random hexamers for priming. Tenfold serial dilutions of plasmid containing the fragment of the HIV-1 genome, ranging from 1 to 10^7 HIV-1 copies per tube, were used for construction of the HIV-1 RNA calibration curve. Tenfold serial dilutions of plasmid containing the fragment of the human RNA polymerase IIα gene, ranging from 1 to 10^7 RNA polymerase Iiα copies per tube, were used for construction of the RNA polymerase IIα calibration curve. We normalized the RNA copies used in the reaction according to the determined number of RNA polymerase IIα copies. The reactions were performed as described for quantitative PCR with primers specific for the R (forward primer; 5'GGGTCTCTCTGGTTAGA3') and

the U5 (reverse primers; 5’ GGGTTCCCTAGTTAGCC 3’; 5’ CTGCTAGAGATTTTCCA CACTGAC 3’) sequences of the HIV cDNA and with a probe specific for the R region (5’ FAM-CAGATCTGAGCCTGGGAGCTCTC-TAMRA 3’).

Inverse PCR. We purified the H12 cell line DNA using the DNeasy Tissue Kit (Qiagen, France) and cleaved it with *Hin*dIII restriction enzyme, which cuts once within the U5 of HIV-based vector LTR. Cleaved DNA was self-ligated and amplified by means of nested PCR in a 50-µl reaction mixture containing 50 mM Tris-HCl (pH 9.2), 1.75 mM $MgCl_2$, each dNTP at 350 µM, and each primer at 45 pmol: UL (5'-GATCAAGGATATCTTGTCT TCGT-3', nt 23 to 45, antisense), UR (5'-CTTTTTGCCTGTACTGGGTCTC-3', nt 438 to 459, sense), IL (5’-TCTTGTCTTCGTTGGGAGTGA-3’, nt 14 to 34, antisense) and IR (5’-GAACCCACTGCTTAAGCCTC-3’, nt 506 to 525, sense). PCR was performed for 40 cycles at 95°C for 60 s, 58°C for 60 s, and 72°C for 180 s. Amplification products were cloned in the pGEM-T-Easy Vector System (Promega, Madison, WI) and sequenced. We used Human BLAT Search (http://genome.ucsc.edu/cgi-bin/hgBlat) to identify the human genome sequence flanking the HIV-1 LTR.

Cell number distribution per well during cloning of H12 cells. Sublimit dilutions of H12 cells were distributed into 96-well microtiter plates to inoculate on average one out of ten wells with one cell. The probability that a cell clone originated from a single cell was estimated according to the formula $f(k;\lambda) = \lambda^k e^{-\lambda} (k!)^{-1}$, where λ is the proportion of wells with outgrown clones and k values are the cell counts that were hypothetically deposited in the microtiter well. The probability for k=0 (no cell per well) and k=1 (one

cell per well) with λ=0.105 is equal to 99.4%, suggesting that with a high probability the clones were derived from one cell. Subcultures were further expanded.
