# Supplementary material for: CpG Methylation Controls Reactivation of HIV from Latency
Source: PLoS Pathog. 2009 Aug 21;5(8):e1000554. doi: 10.1371/journal.ppat.1000554 (PMC2722084; doi:10.1371/journal.ppat.1000554)
Supplement: Table S1 — Number of proviral copies in latently transduced Jurkat clonal cell lines. (0.03 MB PDF) [file ppat.1000554.s002.pdf]

Supplemental Table S1. Number of proviral copies in latently transduced Jurkat clonal cell lines.

Clonal cell line[a]	Number of proviral copies per cell[b]
A2	1.1 ± 0.2
A8	1.1 ± 0.2
G10	1.1 ± 0.2
H12	1.2 ± 0.1
JNLGFP	5.3 ± 1.1

[a] Clonal cell lines latently infected with an LTR-Tat-IRES-EGFP HIV-1–derived vector (A2, A8, G10, H12) and with complete HIV-1 (JNLGFP).

[b] Determined by quantitative PCR, means ± SEM.
