# Supplementary material for: CpG Methylation Controls Reactivation of HIV from Latency
Source: PLoS Pathog. 2009 Aug 21;5(8):e1000554. doi: 10.1371/journal.ppat.1000554 (PMC2722084; doi:10.1371/journal.ppat.1000554)
Supplement: Table S2 — Flanking sequences of HIV-1-based vector integrated in the first intron of the ubiquilin gene in the cell line H12. (0.03 MB PDF) [file ppat.1000554.s003.pdf]

Supplemental Table S2. Flanking sequences of HIV-1-based vector integrated in the first intron of the *ubiquilin* gene in the cell line H12[a].

5' flanking sequence	5' AGGTAAAACAGATTTTTTAAAAATCAGTTGTTTATATT 3'
3' flanking sequence	5' GGCTGTTGTAAATTGATTATGGAAAATTTTGTTCATAC 3'

[a] Determined by sequencing of inverse PCR product (*see* Supplemental Methods).
