# Supplementary material for: CpG Methylation Controls Reactivation of HIV from Latency
Source: PLoS Pathog. 2009 Aug 21;5(8):e1000554. doi: 10.1371/journal.ppat.1000554 (PMC2722084; doi:10.1371/journal.ppat.1000554)
Supplement: Table S3 — Antiretroviral therapy in patients without detectable plasma viremia (<50 copies/ml). (0.05 MB PDF) [file ppat.1000554.s004.pdf]

Supplemental Table S3. Antiretroviral therapy in patients without detectable plasma viremia (<50 copies/ml)[a].

Patient no.	Therapy period: Start	End	HIV inhibitors[b]
4	**April, 2001**	**Up to now**	**3TC, ABV, NVP**
	March, 2001	April, 2001	3TC, ABV, EFV
	March, 2000	March, 2001	AZT, 3TC, IDV, RTV
	April, 1997	March, 2000	AZT, 3TC, IDV
	July, 1992	April, 1997	ddI
9	**June, 2005**	**Up to now**	**3TC, ABV, LPV**
	Dec, 1999	Jun, 2005	d4T, ddI, IDV, RTV
	April, 1997	Dec, 1999	d4T, ddI, IDV
	Oct, 1996	April, 1997	AZT, 3TC
10	**Feb, 2006**	**Up to now**	**FTC, TDF, NVP**
	June, 2001	Feb, 2006	AZT, ddI, EFV
	Jan, 2000	Jun, 2001	AZT, ddI, IDV, RTV
	July, 1997	Jan, 2000	AZT, ddI, IDV
	March, 1997	July, 1997	AZT, ddI, RTV
14	**Jan, 2003**	**Up to now**	**SQV, LPV, RTV**
	Before Jan. 2003		AZT, 3TC, LPV, RTV
18	**Jan, 2006**	**Up to now**	**FTC, TDF, APV, RTV**

[a] The present aviremic period lasting for ≥2 years (also shown in Table 1) is highlighted in bold characters.

[b] AZT, Zidovudine; ABV, Abacavir; APV, Fosamprenavir; ddI, Didanosine; d4T, Stavudine; EFV, Efavirenz; FTC, Emtricitabine; IDV, Indinavir; LPV, Lopinavir; NVP, Nevirapine; RTV, Ritanovir; 3TC, Lamvudine; TDF, Tenofovir; SQV, Saquinavir.
