# Supplementary figures and images for: CpG Methylation Controls Reactivation of HIV from Latency
Source: PLoS Pathog. 2009 Aug 21;5(8):e1000554. doi: 10.1371/journal.ppat.1000554 (PMC2722084; doi:10.1371/journal.ppat.1000554)

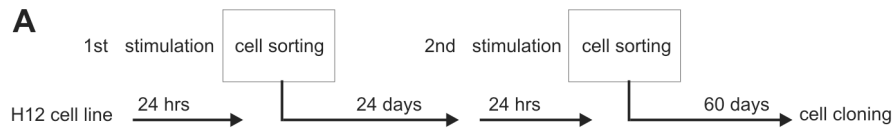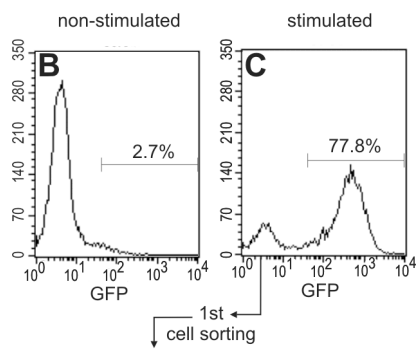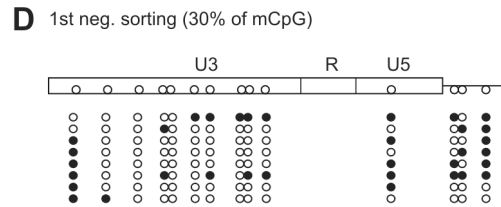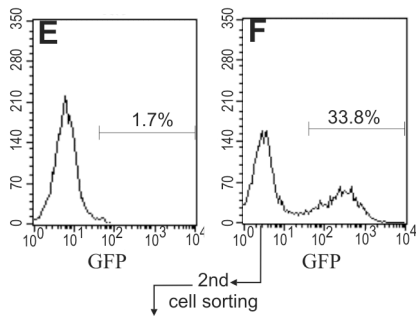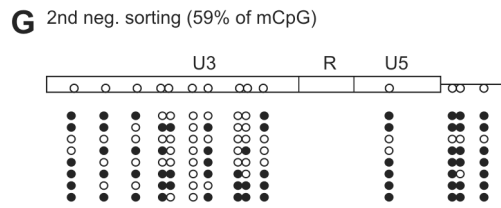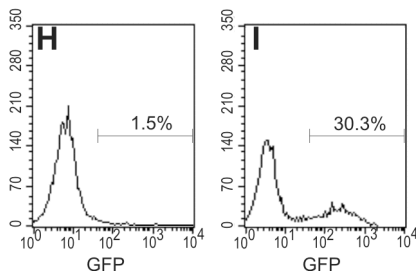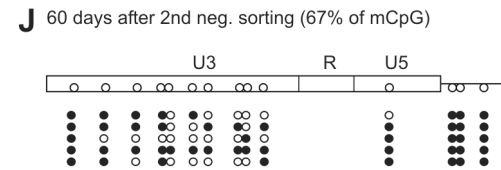

Supplement: Figure S1 — Selection of H12 cells resistant to reactivation of HIV-1 provirus from latency. (A) Flow chart protocol showing activations of H12 cells with TNF-α and PMA, the first and second cell sorting of EGFP-negative cells, and cultivation periods in the absence of exogeneous activator. (B) Immunofluorescence of EGFP in non-activated H12 Jurkat cells. (C) Immunofluorescence of EGFP in H12 cells exposed to TNF-α (10 ng/ml) and PMA (10 nM) for 24 h. (D) CpG methylation profile of the 5′ LTR in H12 cells exposed to TNF-α and PMA for 24 h and negatively sorted for EGFP. (E) Immunofluorescence of EGFP in H12 Jurkat cells activated with TNF-α and PMA for 24 h, negatively sorted for EGFP, and then kept in culture in the absence of exogeneous activator for 24 days. (F) Immunofluorescence of EGFP in H12 Jurkat cells cultured as in (E) and stimulated for the second time with TNF-α and PMA for 24 h. (G) CpG methylation profile of the 5′ LTR in H12 cells cultured as in (F), negatively sorted for the second time for EGFP. (H) Immunofluorescence of EGFP in H12 Jurkat cells cultured as in (F), negatively sorted for EGFP, and then kept in culture in the absence of exogeneous activator for 60 days. (I) Immunofluorescence of EGFP in H12 Jurkat cells cultured as in (H) and stimulated for the third time with TNF-α and PMA for 24 h. (J) CpG methylation profile of the 5′ LTR in H12 cells cultured as in (H). Open circles, nonmethylated CpG residues; closed circles, methylated CpG residues. Bars in B, C, E, F, H, and I represent percentages of EGFP-positive cells. (0.13 MB PDF) [file ppat.1000554.s005.pdf]

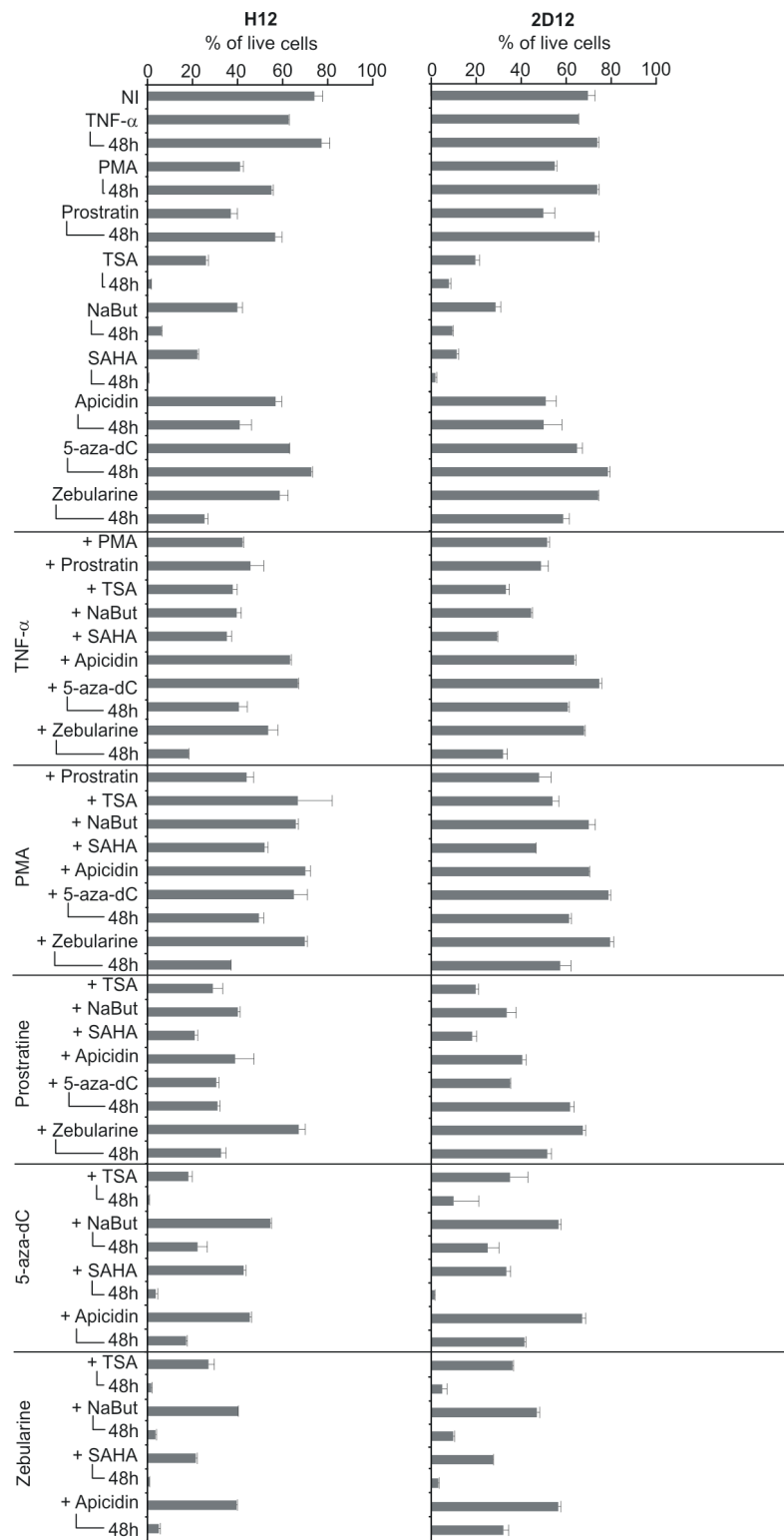

Supplement: Figure S2 — Survival of H12 and 2D12 cells treated with HIV-1 inducers. Percentages of live H12 and 2D12 cells relative to non-induced (NI) control were determined by means of vital staining with Hoechst 33258 and flow cytometry after 24 h and/or 48 h stimulation with the indicated substances. Results that represent duplicates or several independent experiments are presented as means±SD. (0.16 MB PDF) [file ppat.1000554.s006.pdf]
